# Anti-Tumor Effects of MAPK-Dependent Tumor-Selective Oncolytic Vaccinia Virus Armed with CD/UPRT against Pancreatic Ductal Adenocarcinoma in Mice

**DOI:** 10.3390/cells10050985

**Published:** 2021-04-23

**Authors:** Hajime Kurosaki, Motomu Nakatake, Teruhisa Sakamoto, Nozomi Kuwano, Masato Yamane, Kenta Ishii, Yoshiyuki Fujiwara, Takafumi Nakamura

**Affiliations:** 1 Division of Molecular Medicine, Department of Genomic Medicine and Regenerative Therapy, Faculty of Medicine, Tottori University, Yonago 683-8503, Japan; hajime.kurosaki@tottori-u.ac.jp (H.K.); motom.nakatake@tottori-u.ac.jp (M.N.); nozomi.kuwano@gmail.com (N.K.); mt.root.masa81@gmail.com (M.Y.); isiikenta@gmail.com (K.I.); 2 Division of Surgical Oncology, Department of Surgery, Faculty of Medicine, Tottori University, Yonago 683-8504, Japan; tesakamo@tottori-u.ac.jp (T.S.); y-fujiwara@tottori-u.ac.jp (Y.F.)

**Keywords:** oncolytic vaccinia virus, MAPK pathway, antitumor effects, CD/UPRT, prodrug 5-FC, pancreatic cancer

## Abstract

Engineered vaccinia virus serves as an oncolytic virus for cancer virotherapy. We evaluated the oncolytic characteristics of *VGF*- and *O1*-deleted recombinant mitogen-activated protein kinase (MAPK)-dependent vaccinia virus (MDRVV). We found that compared with viruses with the deletion of either gene alone, MDRVV is more attenuated in normal cells and can replicate in cancer cells that exhibit constitutive ERK1/2 activation in the MAPK pathway. We armed MDRVV with a bifunctional fusion gene encoding cytosine deaminase and uracil phosphoribosyltransferase (CD/UPRT), which converts 5-fluorocytosine (5-FC) into chemotherapeutic agents, and evaluated its oncolytic activity alone or in combination with 5-FC in human pancreatic cancer cell lines, tumor mouse models of peritoneal dissemination and liver metastasis, and ex vivo-infected live pancreatic cancer patient-derived tissues. CD/UPRT-armed MDRVV alone could efficiently eliminate pancreatic cancers, and its antitumor effects were partially enhanced in combination with 5-FC in vitro and in vivo. Moreover, the replication of MDRVV was detected in tumor cells of patient-derived, surgically resected tissues, which showed enlarged nuclei and high expression of pERK1/2 and Ki-67, and not in stromal cells. Our findings suggest that systemic injections of CD/UPRT-armed MDRVV alone or in combination with 5-FC are promising therapeutic strategies for pancreatic ductal adenocarcinoma.

## 1. Introduction

Vaccinia virus (VV), a double-stranded DNA virus of the family Poxviridae, is best known for its use as a smallpox vaccine until the 1970s. Recently, VV has been shown to kill cancer cells in a variety of ways, including through lysis following viral infection and subsequent replication, triggering antitumor immune responses, and disrupting the tumor-associated vasculature [1,2]. Its preclinical and clinical applications for cancer therapy have been facilitated by the genetic modification of the VV genome [1,2].

The most advanced VV immunotherapeutic agent, Pexa-Vec (JX-594), has been engineered by deleting the viral gene that encodes thymidine kinase (TK) for tumor selectivity through insertion and expression of the immuno-stimulating gene that encodes granulocyte/macrophage-colony-stimulating factor (GM-CSF), to enhance antitumor immune responses via dendritic cell activation and the subsequent stimulation of T-lymphocyte activity [3]. Its efficacy as an intratumoral therapeutic agent in combination with sorafenib is being evaluated in phase III trials against hepatocellular carcinoma (ClinicalTrials.gov Identifier: NCT02562755). As tumor and not normal cells express abundant TK, deleting the viral *TK* gene is compensated by cellular TK and does not impair therapeutic replication in cancer cells. In contrast, viral *TK* deletion inhibits pathogenic viral replication in normal cells [1,2,3].

Furthermore, tumor-selective viral replication is enhanced by the deletions of both *TK* and another gene on the VV genome, which encodes the vaccinia growth factor (VGF) [4] and is the viral analogue of cellular epidermal growth factor (EGF) [5,6]. Deletion of the *VGF* gene alone decreases pathogenic viral replication, but not completely [7]. VGF is a secreted protein, produced early in a viral infection, which has the paracrine role of inducing mitogens to prime neighboring uninfected cells for vaccinia infection, as well as the autocrine role of substantially contributing to efficient viral replication by activating the EGFR-dependent mitogen-activated protein kinase (MAPK)-ERK pathway [8,9,10]. The O1 protein helps to sustain the extracellular signal-regulated kinase 1/2 (ERK1/2) signaling initiated by VGF [11]. It is hypothesized that the O1 protein might be responsible for residual viral pathogenicity, which was observed in mice treated with the *VGF*-deleted VV.

Pancreatic ductal adenocarcinomas (PDACs) are aggressive cancers that have the lowest 5-year survival rates (9% for all stages and only 3% for metastatic disease) among all cancers [12]. Despite advances in surgical approaches and various chemotherapy regimens, current therapeutics are insufficient to improve survival rates. On the other hand, earlier prognosis has not been realized for decades, and more than half of the PDAC patients are diagnosed at an advanced stage [12]. Therefore, new therapeutic and diagnostic methods for PDAC are urgently required. PDAC has been characterized by four commonly mutated major genes, *KRAS*, *TP53*, *CDKN2A*, and *SMAD4* [13,14], which are ideal therapeutic targets. Particularly, the *KRAS* mutation (mainly G12) in the MAPK pathway has been observed in 90% of all PDAC cases [15,16,17,18]. Constitutively activated *KRAS* not only increases endogenous expression of the upstream protein EGFR and induces its hyperactivation [19,20] but also triggers downstream signaling in the RAF/MEK/ERK pathway [21]. Small molecules that have been designed to bind irreversibly and specifically to these targets in the MAPK pathway, such as erlotinib [22] and selumetinib [23], are under evaluation as therapeutic agents in clinical trials.

It is expected that MAPK-dependent recombinant vaccinia virus (MDRVV) with deletions in both *VGF* and *O1* would be more highly attenuated in normal cells and could replicate in tumor cells that exhibit constitutive ERK1/2 activation in the MAPK pathway due to oncogenic mutations in the *EGFR*, *RAS*, and *Raf* genes [24,25,26]. This aberrant MAPK activation in PDAC strongly encourages us to apply MDRVV to PDAC treatment. Furthermore, we armed MDRVV with a suicide gene in the form of a bifunctional fusion gene encoding yeast cytosine deaminase (CD) and uracil phosphoribosyltransferase (UPRT), which converts the nontoxic 5-fluorocytosine (5-FC) into the chemotherapeutic 5-fluorouracil (5-FU) and subsequently into 5-fluorouracil-monophosphate, which inhibits DNA and protein synthesis [27]. This suicide and prodrug system has been utilized for other oncolytic viruses such as vvDD-CD [28], Toca 511 [29], MV-CD-antiEGFR [30], and TG6002 [31], and its therapeutic potential has been enhanced through a combination with 5-FC without severe side effects in preclinical and clinical investigation. 5-FU is a chemotherapeutic agent used in patients with advanced pancreatic cancer [32]. If MDRVV achieves tumor-selective replication, it is expected that CD and UPRT expression will be restricted to tumor tissues and will enhance the therapeutic index in combination with 5-FC.

In this study, we established two tumor mouse models of peritoneal dissemination and liver metastasis using six types of human PDAC cell lines, including CD44v9-positive cancer stem cells (CSCs) [33]. Since MDRVV and PDAC cells express *Renilla* and firefly luciferases, respectively, viral distribution and tumor growth can be non-invasively visualized via the administration of the respective substrates. By using these models, we evaluated the tumor-selective replication, oncolytic activity, and safety of MDRVV, as well as the antitumor effects of CD/UPRT-armed MDRVV in combination with 5-FC. The therapeutic availability was also validated in pancreatic cancer patient-derived surgically resected tissues as a preclinical evaluation.

## 2. Material and Methods

### 2.1. Cell Lines

The human PDAC cell lines AsPC-1, BxPC-3, Panc 10.05, SW 1990, MIA PaCa-2, and PANC-1 were purchased from the American Type Culture Collection (Manassas, VA, USA). The cells were grown in the appropriate medium (Wako, Osaka, Japan) with 10% fetal bovine serum (FBS), except for Panc 10.05 cells (15% FBS and 10 U/mL human insulin), at 37 °C in a humidified atmosphere with 5% CO_2_. The normal human lung fibroblast (NHLF) cells were purchased from Lonza (Tokyo, Japan) and cultured according to the manufacturer’s protocol. For in vivo therapeutic experiments, PDAC cell lines stably expressing *Renilla* luciferase (Rluc) were generated by transducing parental cells using VSV-G pseudotyped lentiviral vectors, as described previously [34]. To generate the lentivectors, 293T cells were cotransfected with the gag-pol expression plasmid pCMVΔ8.91, VSV.G envelope expression plasmid pMD-G, and vector plasmid pHR-SIN-dlNotI encoding hRluc-Neo fusion gene derived from pmirGLO vector (Promega, Madison, WI, USA). The transduced cells were selected with 0.5 (for BxPC-3 and SW 1990), 1 (for AsPC-1, Panc 10.05 and PANC-1), or 1.5 (for MIA PaCa-2) mg/mL Geneticin™ Selective Antibiotic (Thermo Fisher Scientific, Waltham, MA, USA). Further, CSCs that expressed high levels of CD44v9 were sorted from the Rluc-expressing AsPC-1 cells using the anti-human CD44v9 antibody (Cosmo Bio Co., Ltd., Tokyo, Japan) and FACSAria (BD Biosciences, Piscataway, NJ, USA), according to the manufacturer’s protocol.

### 2.2. Virus Preparation

Recombinant VVs were constructed using the LC16mO strain [35], as reported [36,37]. As described in our previous study [38], VGF+/O1+ or VGF−/O1− (LG-DsRed) VVs were developed by deleting the viral genes encoding hemagglutinin (*HA*) locus or the *VGF* and *O1* loci, respectively, and inserting the expression cassette composed of a luciferase-enhanced green fluorescent protein (LG) encoding fusion gene under a synthetic vaccinia virus promoter (SP) [39] or Discosoma sp. red fluorescent protein (DsRed) from the p7.5 promoter. In this study, VGF−/O1+ or VGF+/O1−VVs were generated in CV-1 (ATCC CCL-70) or RK13 (ATCC CCL-37) cells via inserting the cassette expressing the LG encoding fusion gene into the *VGF* or *O1* locus of the parental virus LC16mO genome. Likewise, the *Saccharomyces cerevisiae* CD-UPRT fusion gene (InvivoGen, San Diego, CA, USA) and LG encoding fusion gene under SP were inserted into the *VGF* and *O1* loci of LC16mO genome, resulting in VGF−/O1−VV (CD/UPRT-LG). All viruses were propagated in A549 (ATCC CCL-185) or RK13 cells. Furthermore, LG-DsRed and CD/UPRT-LG viruses were purified for in vivo experiments and dialyzed using OptiPrepTM (Axis-Shield, Oslo, Norway) and a Slide-A-Lyzer™ Dialysis Cassette (Thermo Fisher Scientific, Waltham, MA, USA) according to the manufacturer’s protocol. They were then titrated by the standard plaque assay with RK13 cells and stored at −80 °C until just before use.

### 2.3. Viral Infection

NHLF or AsPC-1 cells were infected with VVs at a multiplicity of infection (MOI) of 1 plaque-forming unit (PFU)/cell in Opti-MEM medium (Life Technologies, Carlsbad, CA, USA) for 1 h at 37 °C in a 24-well plate. Next, the virus suspension was replaced with respective cell media in the presence or absence of serum and cultured for 30 h at 37 °C. Subsequently, cells were imaged under a phase-contrast and a fluorescence microscope (Olympus Corporation, Tokyo, Japan). EGFP fluorescence was quantified based on intensity and area using a Hybrid Cell Count (Keyence, Osaka, Japan). Simultaneously, the endogenous phosphorylated p44/42 MAPK protein (Erk1/2) levels were detected in the NHLF and AsPC-1 cells using the Pierce ERK1/2 Colorimetric In-Cell ELISA Kit (Thermo Fisher Scientific, Waltham, MA, USA).

### 2.4. Cytotoxicity Assay

Pancreatic cancer cell lines were infected with LG-DsRed or CD/UPRT-LG recombinant viruses (AsPC-1: MOI of 0.1; AsPC-1h: MOI of 0.1; BxPC-3: MOI of 0.01 and 0.1; MIA PaCa-2: MOI of 0.1; PANC-1: MOI of 0.01 and 0.1; Panc 10.05: MOI of 0.1; SW 1990: MOI of 0.01 and 0.1). After 48 h of viral infection, 0, 1, 10, or 100 μg/mL of 5-FC (Abcam, Cambridge, UK) was added into each culture media. After 72 h (120 h after initial virus infection), cell viability was assessed using the CellTiter 96 Aqueous Non-Radioactive Cell Proliferation Assay (Promega, Madison, WI, USA).

### 2.5. In Vivo Experiments

The protocols for the following animal experiments were approved by the Animal Experiment Committee of Tottori University, Japan. In the first in vivo experiment, 6-week-old female CB-17/Icr-severe combined immunodeficiency (SCID)/SCID Jcl mice (Charles River Laboratories, Yokohama, Japan) were administered intraperitoneal (I.P.) injections of a single dose of VGF+/O1+, VGF+/O1−, VGF−/O1+, or VGF−/O1− (1 × 10^6^ PFU in 200 μL of phosphate-buffered saline (PBS), pH 7.4, per mouse) virus on day 0 (n = 2 for each group). Next, changes in body weights of the mice were observed for 48 weeks. Additionally, I.P. injections of BxPC-3 cells stably expressing Rluc (5 × 10^6^ cells in 200 μL PBS) were administered to 6-week-old female SCID mice on day 0. On day 8, the mice received a single I.P. injection of VGF+/O1+, VGF+/O1−, VGF−/O1+, or VGF−/O1− (1 × 10^6^ PFU in 200 μL PBS per mouse) virus (n = 5 for each group). Control animals (mock therapy) were injected with 200 μL PBS without any virus (n = 4). The mice were euthanized at the end of the experiment or when any of the following occurred: signs of severe viral toxicity such as pock lesions on body surfaces and weight loss >30%.

In the second in vivo experiment, 6-week-old female SCID mice were injected I.P. with cells from six Rluc-expressing human pancreatic cancer cell lines (5 × 10^6^ cells in 200 μL PBS). After 1 to 2 weeks, 200 μL PBS and 1 × 10^6^ PFU CD/UPRT-LG viruses diluted in 200 μL PBS were injected I.P. into control and experimental mice, respectively (n = 4 or 3 for Panc 10.05 group or other groups, respectively). Tumor growth and viral replication were non-invasively detected by firefly luciferase (Fluc) or Rluc luminescence, respectively, following the I.P. injections of 150 μL of ViviRen In Vivo *Renilla* Luciferase Substrate (18.5 μg/mouse; Promega, Madison, WI, USA) on days −2 and 11 or 200 μL VivoGlo Luciferin, In Vivo Grade (3 mg/mouse; Promega, Madison, WI, USA) on days 2 and 10 after virus injection. Bioimaging was carried out using NightSHADE LB985 (Berthold Technologies, Bad Wildbad, Germany), and mice were anesthetized with isoflurane during the detection. Fluc or Rluc luminescence was quantified according to the manufacturer’s protocol.

In the third in vivo experiment, a small upper-quadrant incision was made under isoflurane anesthesia to expose the spleen of each 6-week-old female athymic nude mouse (Charles River Laboratories, Yokohama, Japan), and AsPC-1h cells (1 × 10^6^ cells in 200 μL PBS) were directly implanted into the spleen, and the incision was closed using 6-0 nylon surgical sutures. After 3 weeks of tumor transplantation, tumor growth, and metastasis were confirmed by Rluc imaging as described previously herein. Regarding the selection of injection routes, the mice (n = 6 for each group) received either a single I.P. or intravenous (I.V.) injection of LG-DsRed via 5 × 10^7^ PFU in 200 μL of PBS. Control animals (mock therapy) were injected with the same volume of PBS. However, in the case of 5-FC combination treatments, mice were first administered I.V. injections of PBS or 1 × 10^7^ PFU of CD/UPRT-LG at 3 and 4 weeks after tumor transplantation (on days 0 and 8). Next, 6 days after the second virus injection, mice were injected I.P. with PBS or 12.5 mg 5-FC (Abcam, Cambridge, UK) every day for 3 weeks (from days 14 to 36 except on days 21 and 29). The tumor growth and viral distribution were evaluated by noninvasive imaging and quantified as described previously herein. The mice were euthanized at the end of the experiment or when any of the following occurred: signs of severe viral toxicity such as pock lesions on body surfaces and weight loss >30%.

### 2.6. Ex Vivo Infection and Immunohistochemical Analysis

Tissues were obtained from patients after providing informed consent during the resection of PDACs at the Tottori University Hospital, Japan. Tissue samples were punched, cut out, and cultured in Opti-MEM (Life Technologies, Carlsbad, CA, USA) medium supplemented with 10% FBS and penicillin-streptomycin (Wako, Osaka, Japan). After 24 h of incubation, 1 × 10^6^ PFU of LG-DsRed viruses were added into the culture media and incubated for 120 h. Formalin-fixed, paraffin-embedded tissue sections were treated to remove the paraffin and unmask the antigen epitopes for detection. After deparaffinization, antigen activation treatment was carried out by the microwave method using 10 mM sodium citrate buffer (pH 6.0), and serial sections were blocked with a blocking solution (tris-buffered saline with tween 20/5% normal goat serum) at room temperature for 1 h. The sections were then incubated with anti-GFP antibody (CST, Tokyo, Japan), anti-phosphorylated p44/42 MAPK protein (Erk1/2) antibody (CST, Tokyo, Japan), or anti-Ki-67 antibody (CST, Tokyo, Japan) at 4 °C overnight. Next, diaminobenzidine was used as a chromogen following the manufacturer’s protocol for the SignalStain Boost IHC Detection Reagent (CST, Tokyo, Japan) and SignalStain DAB Substrate Kit (CST, Tokyo, Japan), and cells were counterstained with hematoxylin (Wako, Osaka, Japan).

### 2.7. Statistical Analysis

The difference in pERK1/2 expression was analyzed for statistical significance using one-way analysis of variance (ANOVA) and the Bonferroni test when ANOVA showed overall significance. Cytotoxicity assays and viral Fluc luminescence in liver metastasis mouse tumor model were evaluated using the two-tailed unpaired *t*-test. P-values less than 0.05 were considered statistically significant. Survival curves were constructed using the Kaplan–Meier method. Survival times were statistically analyzed by the log-rank test. Data were analyzed using GraphPad Prism version 5 (GraphPad Software, San Diego, CA, USA).

## 3. Results

### 3.1. Tumor Specificity of VGF−/O1−VV Is Dependent on Cellular MAPK/ERK1/2 Activity

**Figure 1:**
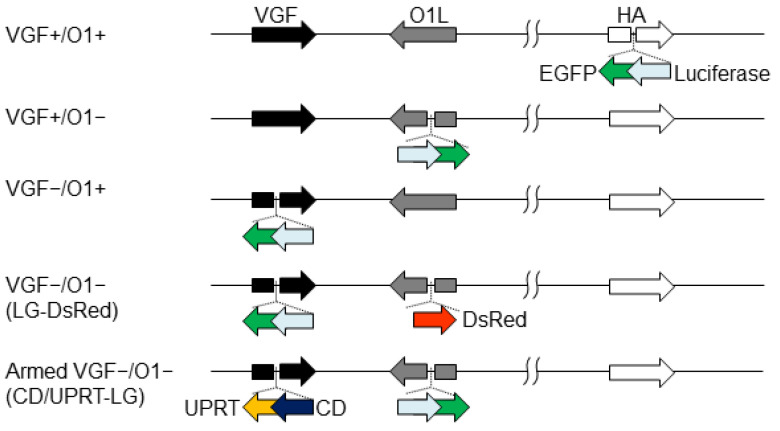
Recombinant vaccinia viruses genetically engineered by the deletion of viral genes and expression of transgenes. Vaccinia growth factor (*VGF*)- and/or *O1*-deleted viruses were generated through the insertion of a gene cassette expressing luciferase-fused EGFP (LG) and/or DsRed into the *VGF* and *O1* gene loci. *VGF*- and *O1*-deleted armed virus was developed via the insertion of a gene cassette expressing cytosine deaminase-fused uracil phosphoribosyl transferase (CD/UPRT) and LG into the *VGF* and *O1* gene loci, respectively. *VGF*- and *O1*-intact control viruses were constructed through the insertion of a gene cassette expressing LG into the *HA* gene loci.

The expression cassette encoding luciferase and EGFP (LG) was inserted into the *HA*, *O1*, or *VGF* locus of an LC16mO strain of VV, resulting in VGF+/O1+, VGF+/O1−, or VGF−/O1+VV, respectively. Insertion into the *HA* gene did not affect the virus growth ability of LC16mO, which was used as a control virus. Furthermore, the expression cassette encoding DsRed was inserted into the *O1* locus of the VGF−/O1+VV, resulting in VGF−/O1−VV (LG-DsRed) (Figure 1).

**Figure 2:**
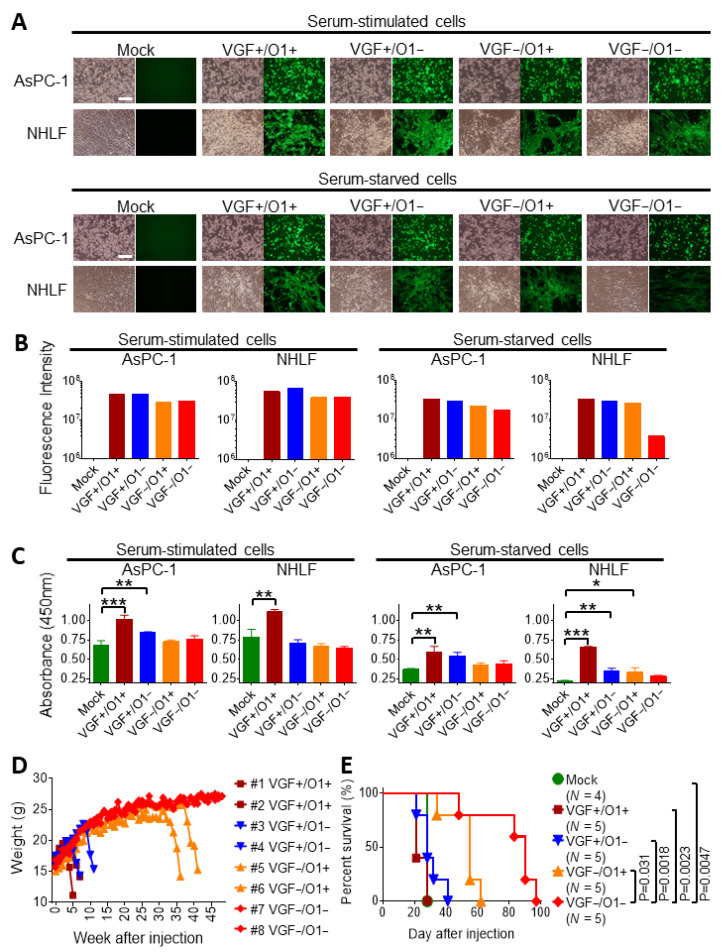
Vaccinia growth factor (*VGF*)- and/or *O1*-deleted mitogen-activated protein kinase-dependent recombinant vaccinia viruses (MDRVVs) are highly attenuated in normal cells but retain their therapeutic replication. The human pancreatic cancer cell line (AsPC-1) or normal human lung fibroblast (NHLF) cells were infected with each recombinant virus at a multiplicity of infection (MOI) of 1 under serum stimulation or starvation conditions, and were cultured for 30 h. (**A**) Bright-field (left) and EGFP (right) representative images of AsPC-1 and NHLF cells are shown. Scale bar, 500 μm. (**B**) The fluorescence intensity in EGFP images of (**A**) was measured using Hybrid Cell Count (n = 1). (**C**) The levels of phosphorylated ERK1/2 (pERK1/2) were evaluated. Data are presented as the mean ± SD (n = 3). * *p* < 0.05, ** *p* < 0.01, *** *p* < 0.001 (one-way ANOVA and the Bonferroni test). (**D**) SCID mice were injected intraperitoneally with 1 × 10^6^ plaque-forming units (PFUs) of each recombinant virus, and changes in body weight were observed for 48 weeks (n = 2). (**E**) BxPC-3 cells stably expressing *Renilla* luciferase were injected intraperitoneally into SCID mice on day 0. On day 8, the mice received a single intraperitoneal injection of each recombinant virus (1 × 10^6^ PFU). Survival curves of mice were generated by Kaplan–Meier analysis (n = 4–5). *p* values were derived by log-rank test.

At 30 h after infection, all recombinant viruses equally induced abundant cytopathic effects (CPEs) following EGFP expression in not only human pancreatic cancer cell lines (AsPC-1) during both serum-stimulated and serum-starved conditions, but also in normal human lung fibroblast (NHLF) cells during proliferation induced by serum. In contrast, VGF−/O1−VV exhibited marginal CPEs in serum-starved NHLF cells, although VGF+/O1+VV, VGF−/O1+VV, and VGF+/O1−VV induced moderate CPEs in NHLF cells during serum starvation (Figure 2A). Consistently, viral EGFP expression was dramatically reduced in serum-starved NHLF cells, but not in serum-stimulated NHLF cells (Figure 2B).

Furthermore, these results were correlated with ERK1/2 activity. No significant changes in the levels of phosphorylated ERK1/2 (pERK1/2) were noted in serum-stimulated AsPC-1 and NHLF cells. Additionally, pERK1/2 was still maintained at a higher level in serum-starved AsPC-1 cells than in serum-starved NHLF cells. Thus, the levels of pERK1/2 were relatively similar in both serum-stimulated AsPC-1 and NHLF cells and serum-starved AsPC-1 cells after viral infection compared with those in mock cells. However, pERK1/2 expression was dramatically reduced by serum starvation in NHLF cells. VGF−/O1−VV infection did not increase pERK1/2 levels compared with those in mock-treated serum-starved NHLF cells, whereas VGF+/O1+VV, VGF+/O1−VV or VGF−/O1+VV resulted in a significant increase (*p* < 0.001, *p* < 0.01 or *p* < 0.05, respectively; Figure 2C).

### 3.2. Tumor Specificity of VGF-/O1-VV Enhances Its Therapeutic Index

Changes in body weights were evaluated by administering a single I.P. injection of each virus into SCID mice (Figure 2D). The VGF+/O1+VV or VGF+/O1−VV-administered mice died on days 35/49 or 49/77, respectively, after injection due to rapid weight loss. The VGF−/O1+VV-administrated mice also lost weight and died by days 252 and 287 after injection. On day 16, the VGF+/O1+VV and VGF+/O1−VV viruses spread to several areas of the body, including the tail, paws, and face, where pock lesions were observed, while one of the mice treated with the VGF−/O1+VV exhibited pock lesions on the tail on day 231 (Supplementary Figure S1). In contrast, the VGF−/O1−VV-injected mice survived for 48 weeks without any viral spread or loss of weight.

Moreover, the oncolytic activity was evaluated by a single I.P. injection of each virus (10^6^ PFU) into SCID mice bearing peritoneally disseminated BxPC-3 xenografts (Figure 2E). VGF−/O1−VV administration significantly prolonged their survival rates compared with those after VGF−/O1+VV (*p* = 0.031), VGF+/O1−VV (*p* = 0.0018), VGF+/O1+VV (*p* = 0.0023), and mock (*p* = 0.0047) therapies. The results were supported by intraperitoneal tumor growth and viral distribution, which were detected separately via Rluc and Fluc luminescence, respectively. All recombinant viruses exhibited comparable viral replication in intraperitoneal tumors 3 days after viral injection, and mostly eliminated the tumors by day 11 after viral treatment (Supplementary Figure S2A). Following this, the VGF+/O1+VV or VGF+/O1−VV viruses spread from the tumor to several areas of the body, including the tail, paws, and face, where pock lesions were observed. The VGF−/O1+VV or VGF−/O1−VV viruses did not induce any pock lesions with viral replication on day 10 (Supplementary Figure S2B). Taken together, our study demonstrated that MAPK-dependent oncolytic VGF−/O1−VV enhances the safety profile, tumor specificity, and therapeutic index in SCID mice bearing peritoneally disseminated BxPC-3 xenografts.

### 3.3. In Vitro Viral Oncolytic Effect of CD/UPRT-Armed MDRVV and 5-FC Combination Therapy

**Figure 3:**
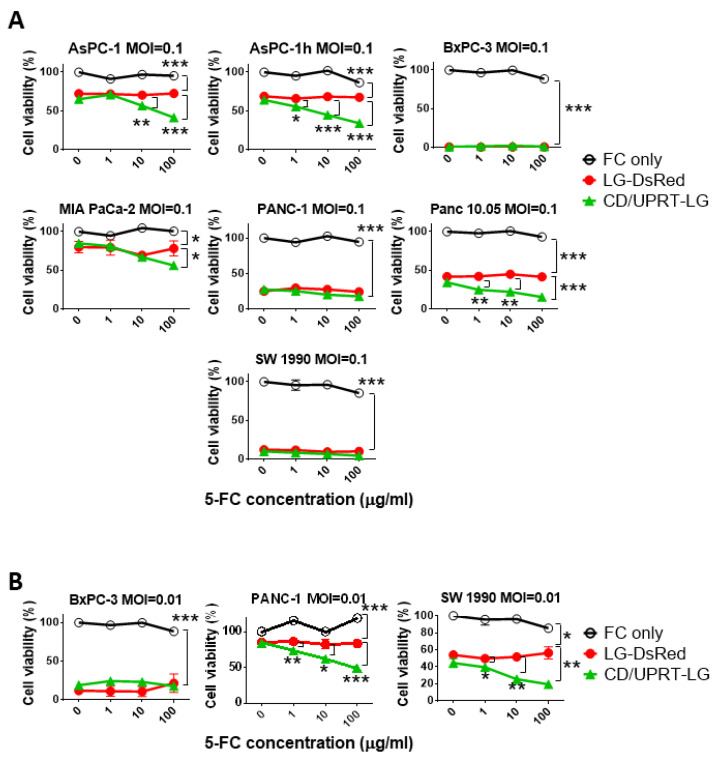
Cell viability tests show cytotoxicity with mitogen-activated protein kinase-dependent recombinant vaccinia virus (MDRVV) alone or in combination with armed MDRVV and 5- fluorocytosine (5-FC) in human pancreatic ductal adenocarcinoma cell lines. (**A**) Pancreatic cancer cell lines were infected with each recombinant virus at a multiplicity of infection (MOI) of 0.1, and 48 h later. Cells were treated with 0, 1, 10, or 100 μg/mL of 5-FC. Cell viabilities were determined 120 h after virus infection. (**B**) BxPC-3, PANC-1, and SW 1990 cells were infected with each recombinant virus at an MOI of 0.01 and combined with 5-FC as described in (**A**). Data are presented as the mean ± SD (n = 3). * *p* < 0.05, ** *p* < 0.01, *** *p* < 0.001 (two-tailed unpaired t-test).

The synergistic effect of recombinant virus and 5-FC was evaluated in pancreatic cancer cells. Six different cell lines were infected with VGF−/O1−VV (LG-DsRed), and the virus was armed with the expression of CD/UPRT (CD/UPRT-LG) at an MOI of 0.1, and 5-FC was added at 48 h after infection. AsPC-1, AsPC-1h, MIA PaCa-2, and Panc 10.05 cells showed synergistic cell death following infection with CD/UPRT-armed virus in a 5-FC dose-dependent manner (Figure 3A). PANC-1 and SW 1990 cells also showed this synergistic effect at lower infection titers (MOI of 0.01; Figure 3B), but BxPC-3 cells were killed by the virus alone because of their low LC_50_ (Supplementary Figure S3). There were no differences in the oncolytic effect of LG-DsRed and the armed CD/UPRT-LG without 5-FC. These synergistic effects were also observed in other virus-oncolysis-resistant cell types such as ovarian OVCAR3 and SKOV3 and colon LoVo and HT-29 cell lines (Supplementary Figure S4).

### 3.4. In Vivo Oncolytic Activity Against Pancreatic Cancers

**Figure 4:**
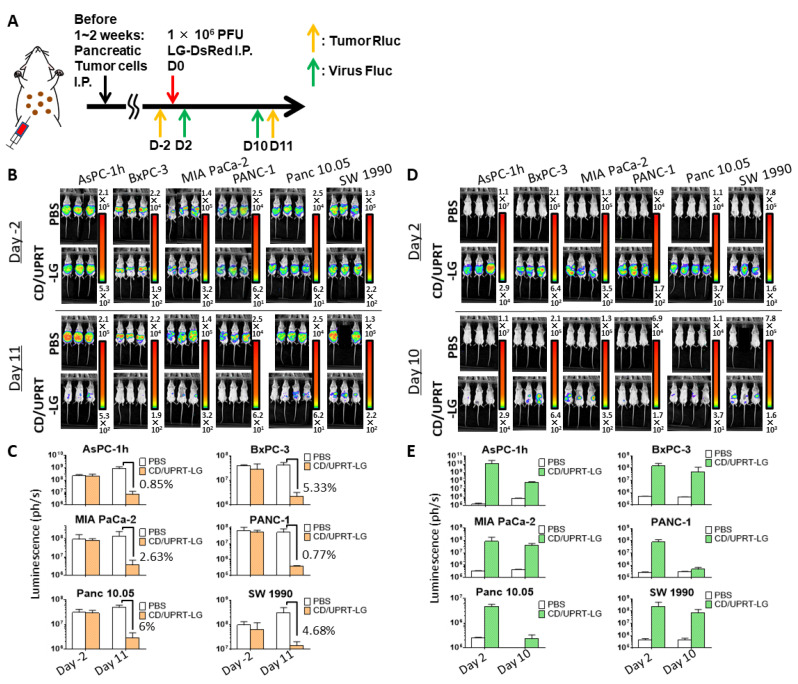
Mitogen-activated protein kinase-dependent recombinant vaccinia virus (MDRVV) (CD/UPRT-LG) efficiently eliminates peritoneally disseminated tumors and its replication decreases following tumor elimination in mouse tumors derived from human pancreatic ductal adenocarcinoma cell lines. (**A**) Schematic representation of the study design and timeline of tumor growth and viral distribution. Severe combined immunodeficiency mice with peritoneally disseminated six pancreatic cancer cell lines expressing *Renilla* luciferase (Rluc) were intraperitoneally injected with CD/UPRT-LG (1 × 10^6^ PFU). (**B**) Tumor Rluc luminescence was detected on day 2 before and on day 11 after virus inoculation. One mouse bearing peritoneally disseminated SW 1990 xenografts died due to tumor burden 9 days after PBS treatment. (**C**) Quantification of tumor Rluc luminescence was determined from (**B**). (**D**) Viral firefly luciferase (Fluc) luminescence was determined on days 2 and 10 after virus inoculation. One mouse bearing peritoneally disseminated SW 1990 xenografts died due to tumor burden 9 days after PBS treatment. (**E**) Quantification of viral Fluc luminescence was determined from (**D**). The data are presented as the mean + SD (n = 2–4).

The oncolytic effects of armed MDRVV (CD/UPRT-LG), without 5-FC, were examined in SCID mice that were injected I.P. with cells from six pancreatic cancer cell lines (AsPC-1h, BxPC-3, MIA PaCa-2, PANC-1, Panc 10.05, and SW 1990). After viral I.P. administration, tumor growth, and viral replication were detected separately via Rluc and Fluc luminescence, respectively (Figure 4A). Rluc luminescence almost disappeared 11 days after virus administration (Figure 4B). All tumors decreased to <6% following virus treatment (Figure 4C). Moreover, the abundant viral Fluc luminescence, which was observed 2 days after virus injection in all virus-treated mice, decreased 8 days later (Figure 4D,E). The reduced viral replication was closely related to more than 94% tumor regression (Figure 4B,C). These results suggest that armed VGF−/O1−VV replicate in tumor tissues but not in normal tissues, similar to the tumor specificity of VGF-/O1-VV observed in mice with and without intraperitoneal tumors (Figures S1 and S2).

### 3.5. Combinational Therapy of Armed MDRVV and 5-FC in Clinically Relevant Mouse Tumor Model

**Figure 5:**
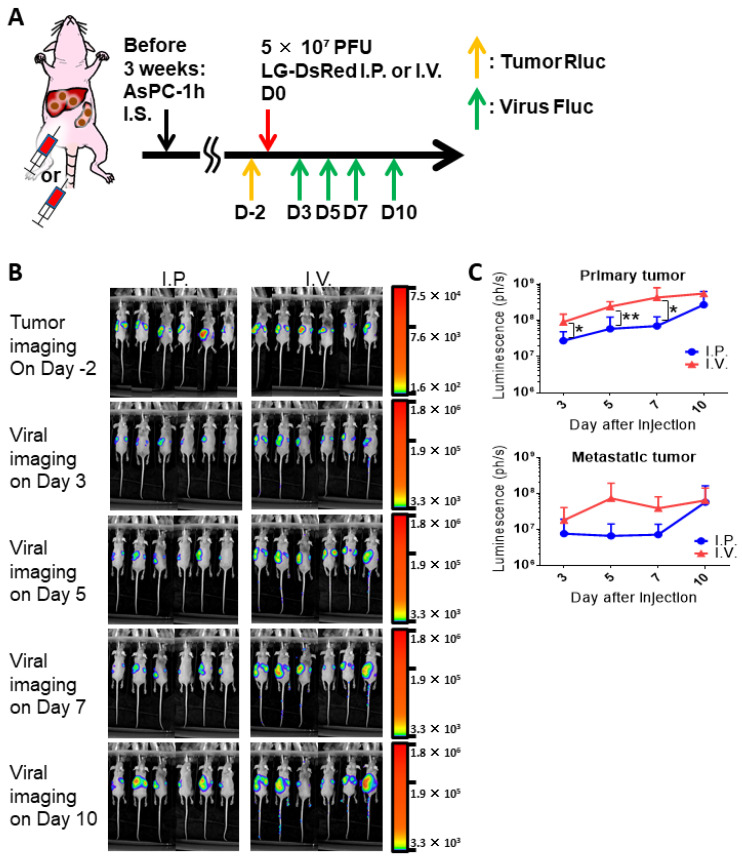
Comparison of intraperitoneal (I.P.) or intravenous (I.V.) injections of mitogen-activated protein kinase-dependent recombinant vaccinia virus (MDRVV) (LG-DsRed) in a mouse model harboring liver metastasis caused by human pancreatic ductal adenocarcinoma cells. (**A**) Schematic representation of the study design and timeline of tumor growth and viral distribution. (**B**) AsPC-1 CD44v9-high cells expressing *Renilla* Luciferase (Rluc; AsPC-1h) were injected intrasplenically into athymic nude mice and liver metastasis was detected via tumor Rluc luminescence on day 2 before viral treatment. Mice were injected intraperitoneally or intravenously with 5 × 10^7^ PFU of MDRVV (LG-DsRed). Viral replication was detected on days 3, 5, 7, and 10 via firefly luciferase (Fluc) luminescence. (**C**) Quantification of viral Fluc luminescence in primary splenic and metastatic liver sites was determined from (**B**). The data are presented as the mean ± SD (n = 6). * *p* < 0.05, ** *p* < 0.01 (two-tailed unpaired *t*-test).

To evaluate the oncolytic effect of the virus alone or in combination with 5-FC, we established a clinically relevant mouse tumor model (Figure 5A) using the fraction of CD44v9-high cells (AsPC-1h) obtained from Rluc-expressing parental AsPC-1 cells (with the highest LC_50_ in pancreatic cancer cells). Tumor Rluc imaging showed that intrasplenic transplantation of AsPC-1h cells resulted in tumor development in not only primary spleens but also resulted in liver metastasis in nude mice (Figure 5B). In the tumor model, we compared virus I.P. or I.V. injections of MDRVV (LG-DsRed). Comparable viral Fluc luminescence was detected in both primary and metastatic tumors on day 3 after I.P. and I.V. injections (Figure 5B). Interestingly, the Fluc luminescence in tumor sites increased more rapidly in I.V.-treated mice from days 5 to 10 than in I.P.-treated mice (Figure 5B). Quantification of Fluc luminescence also indicated that I.V. injection resulted in higher viral replication than that after I.P. injection in both primary spleen and metastatic liver sites (Figure 5C). These results demonstrated that I.V. administration would be better than I.P. injection to reach tumor tissues.

**Figure 6:**
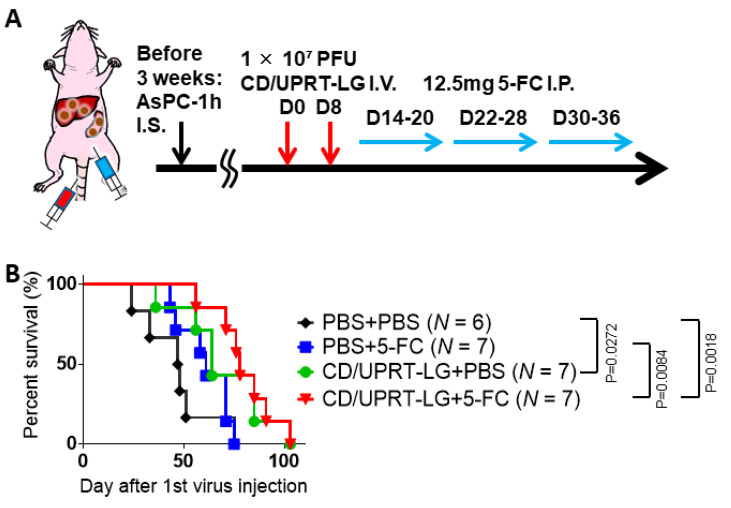
Combination treatment with mitogen-activated protein kinase-dependent recombinant vaccinia virus (MDRVV) (CD/UPRT-LG) and 5-fluorocytosine (5-FC) increases the therapeutic effect in a mouse model harboring liver metastasis caused by human pancreatic ductal adenocarcinoma cells. (**A**) Schematic representation of the study design and timeline of tumor growth and viral distribution. (**B**) The nude mice with liver metastasis were intravenously injected with 1 × 10^7^ PFU of MDRVV (CD/UPRT-LG) on days 0 and 8. Next, 12.5 mg 5-FC was injected intraperitoneally into the mice every day from days 14 to 36, except on days 21 and 29. Survival curves of mice were generated by Kaplan–Meier analysis (*n* = 6–7) and *p* values were determined using the log-rank test.

Thus, the combined effects of armed MDRVV (CD/UPRT-LG) and 5-FC were evaluated by I.V. treatment in the clinically relevant mouse tumor models of PDAC. Comparable tumor Rluc luminescence was detected in both primary and metastatic sites on day 1 before viral treatment in each group (Supplementary Figure S5). The mice were injected I.V. with CD/UPRT-LG twice and then injected I.P. with 5-FC each day of the observation period (Figure 6A). Virus-treated mice (CD/UPRT-LG+PBS) showed significantly prolonged survival compared with the mock-treated group (PBS + PBS; *p* = 0.027), but there was no statistical difference in survival compared with that after 5-FC-only treatment (PBS + 5-FC). However, mice treated with CD/UPRT-LG + 5-FC exhibited significantly prolonged survival rates compared with both the mock and 5-FC-only treatment groups (*p* = 0.0018 and 0.0084, respectively). Thus, the combinational therapy with 5-FC showed a tendency to increase the therapeutic effect of CD/UPRT-armed VV (Figure 6B).

### 3.6. Ex Vivo Infection of Patient-Derived Tumor Tissue

**Figure 7:**
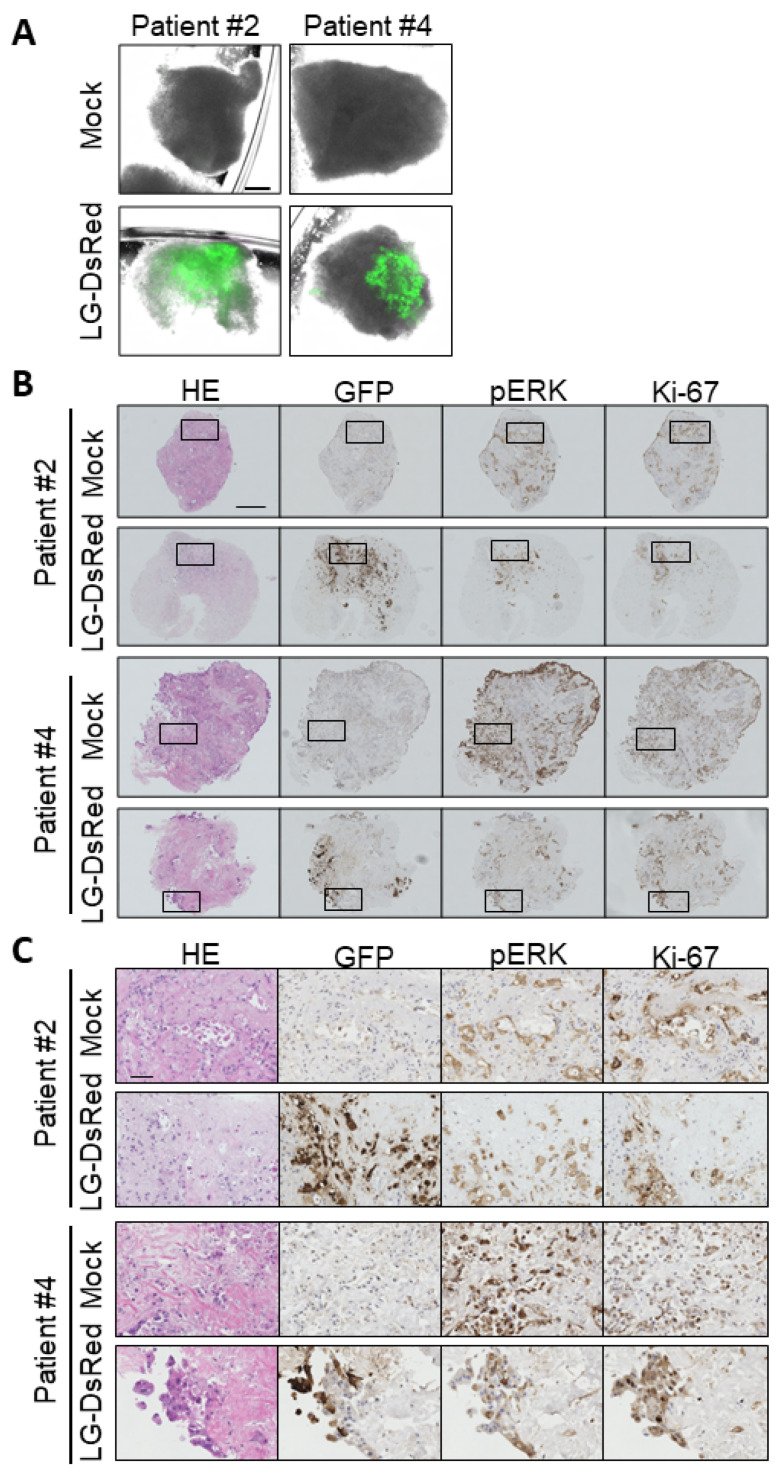
Mitogen-activated protein kinase-dependent recombinant vaccinia virus (MDRVV) achieves tumor-selective replication in tissue cultures from pancreatic ductal adenocarcinoma (PDAC) patients. (**A**) Minced pancreatic tissue samples derived from human pancreatic cancer patients were infected with 1 × 10^6^ plaque-forming units (PFU) of LG-DsRed. Viral GFP was photographed at 120 h after infection (scale bar: 300 μm). (**B**) Immunohistochemical staining of human tissue samples in (**A**). Tissues were stained with hematoxylin and eosin, an anti-GFP antibody, an anti-phosphorylated p44/42 MAPK protein (Erk1/2) antibody, or anti-Ki-67 (scale bar: 300 μm). (**C**) Extended images of those marked with squares in (**B**) (scale bar: 50 μm).

Finally, the replication of VGF−/O1−VV was examined in patient-derived tissue samples. The tumor tissues derived from pancreatic cancer patients were punched out, cut, and infected with LG-DsRed. EGFP expression showing viral infection and replication was detected in surgically resected tissues (Figure 7A); after fixation and embedding, the virus distribution and expression of pERK or Ki-67 were detected by immunohistochemistry staining (Figure 7B). EGFP+ cells had enlarged nuclei and were positive for both pERK and Ki-67 expression, indicating that cells infected with LG-DsRed were pancreatic cancer cells but not stromal cells such as fibroblasts and endothelial cells (Figure 7C). These results suggest that *VGF*- and *O1*-deleted VV has the potential for tumor-selective replication in PDAC patients.

## 4. Discussion

The attenuated, replicating vaccinia virus strain LC16mO used in this study is an attractive backbone for engineering a novel oncolytic agent because it has an extremely low neurovirulence profile. LC16mO was isolated from the Lister strain through LC16, by repeated passages in primary rabbit kidney cells and selecting for their temperature sensitivities [40,41]. The virus was detected from 1 to 3 days post-inoculation in the blood of mice which were intraperitoneally injected with LC16mO, although the viremia was extended from 1 to 7 days when mice were simultaneously administrated with cortisone [41]. The observation is correlated with observations that smallpox vaccination in individuals treated with immunosuppressive medications might be dangerous [42]. However, transient immunosuppression with rapamycin or cyclophosphamide inhibits immune responses to viruses and enhances viral oncolysis [43,44].

The safety of MDRVV with the deletions of two viral genes, *VGF* and *O1,* compared with the parental LC16mO, was evaluated in immunodeficient mice to mimic the clinical phenotype of an immunosuppressed patient. We found that the virus with both genes deleted is more highly attenuated in normal cells than the virus with only one of the genes deleted (Figure 2D). VGF+/O1+VV spread from the abdomen to several areas of the animal body, including the tail, paws, and face, where pock lesions were observed at a much earlier time point. The deletion of *O1* caused a small delay in the viral spread. In contrast, the deletion of *VGF* dramatically reduced viral replication and its spread to normal tissues at an earlier time point, whereas the complementary O1 maintained slower viral replication in normal cells and induced viral toxicity following viral spread at later time points (Supplementary Figure S1). On the contrary, the deletion of both *VGF* and *O1*, which respectively stimulate and sustain activation of the MAPK-ERK pathway, completely inhibited viral toxicity in immunodeficient SCID mice. These results strongly suggest that VGF- and O1-mediated activation of the MAPK-ERK pathway is required for viral replication in normal cells and correlates with viral pathogenicity.

Importantly, the deletions of both *VGF* and *O1* did not impair the oncolytic activity in the human pancreatic cancer cell lines (Figure 3) and the tumor mouse models of peritoneal dissemination (Figure 2E and Figure 4) and liver metastasis (Figure 5 and Figure 6). The PDAC cell lines used in this paper had genomic mutations in one or more genes, including *KRAS*, *TP53*, *CDKN2A*, and *SMAD4* according to ATCC^®^ Cell lines and as reported by Deer et al. [45]. AsPC-1, PANC-1, Panc 10.05, SW 1990, and MIA PaCa-2 cells have typical *KRAS* mutations (G12D and G12C), whereas BxPC-3 cells do not have these. When the sensitivity to MDRVV was determined based on the median lethal dose (LD_50_) of the virus/cell (MOI), BxPC-3 cells showed the lowest MOI among these PDAC cell lines, which had the highest sensitivity to MDRVV (Supplementary Figure S3). However, all tumors were dramatically decreased to <6% after I.P. virus treatment in PDAC-affected tumor mouse models (Figure 4).

Furthermore, we confirmed the relationship between in vivo pERK expression and viral replication of MDRVV in the mouse model bearing the peritoneal dissemination of BxPC-3. Mice were sacrificed 3 days after MDRVV treatment and viral replication was observed in all disseminated tumor sites, but not in normal tissues. Moreover, immunohistochemical analysis demonstrated that MDRVV was only detected in tumor cells with the activated MAPK-ERK pathway (Supplementary Figure S6). The BxPC-3 cells harbor *TP53*, *CDKN2A*, and *SMAD4* mutations. SMAD4 loss is associated with the constitutive activation of the ERK and Wnt/β-catenin signaling pathways [46]. Moreover, restoration of SMAD4 in BxPC-3 cells attenuates cell proliferation in vivo [47]. This phenomenon was also observed in our previous studies wherein oncolytic VV with deletions of both *VGF* and *O1* genes achieved tumor-selective replication in mouse models with xenograft tumors derived from ovarian carcinoma with wild-type KRAS [38,48]. These results suggest that not only *KRAS* but other mutations as well enable tumors to acquire a metabolically active cellular environment that supports viral replication and the spread of MDRVV.

Systemic injection of armed MDRVV (CD/UPRT-LG) achieved tumor-selective replication (Figure 5) and exerted antitumor effect in the tumor mouse model of liver metastasis developed using CD44v9-high cells (AsPC-1h) obtained from parental AsPC-1 cells, and the combinational therapy with 5-FC showed a tendency to increase the therapeutic effect of CD/UPRT-armed VV (Figure 6). However, there was no significant difference in antitumor efficacy in mice between CD/UPRT-LG alone and that in combination with 5-FC. CD44 was identified as an important CSC marker involved in high tumorigenesis, drug resistance, and metastasis in various types of tumors, including PDAC [49]. We confirmed that transplantation of the variant isoform CD44v9-high AsPC-1h was more efficient for developing liver metastasis in mice than that with the parental AsPC-1 cells (data not shown). CD44v9 contributes to increased 5-FU resistance in gastric cancer [50] and is related to the increased expression of multidrug resistance protein 1 (MDR1) in pancreatic cancer [33]. Although no changes were observed in the synergistic effect of the armed virus and 5-FC between AsPC-1 and AsPC-1h cells in vitro (Figure 3A), AsPC-1h tumors have the potential to become more resistant to chemotherapeutic agents converted from 5-FC by CD/UPRT-LG. Therefore, the synergistic effect against PDAC might also be enhanced through the inhibition of cystine-glutamate antiporter, which improves the drug resistance induced by CD44v9 expression [50].

The antitumor activity of MDRVV was assessed in an immunodeficient nude mice bearing xenograft tumors in this study. This model enables the evaluation of both oncolytic activity and viral toxicity simultaneously. However, it fails to develop oncolytic virus-elicited antitumor immune responses, which play a critical role in the efficacy of oncolytic virotherapy. Our previous studies demonstrated that *VGF* and *O1*-deleted oncolytic VVs elicited potent antitumor effects via systemic antitumor activity rather than local viral oncolytic activity in syngeneic tumor mouse models using melanoma, colon, and lung carcinomas, and its antitumor effects were enhanced by arming VV with the expression of IL-12 and IL-7 [51] or induction of cell-cell fusion [52]. Thus, it is worth evaluating the combinational effects of armed MDRVV and 5-FC in syngenic mouse tumor models in the future. In addition, I.V. administration of armed MDRVV resulted in higher viral replication in the tumors than that after I.P. administration. The more effective I.V. delivery enhances the synergistic antitumor activity of CD/UPRT-LG in combination with 5-FC. Pretreatment with PI3Kδ-selective inhibitors before intravenous delivery of VV has the potential to improve viral delivery to tumors via the inhibition of viral attachment to systemic macrophages [53]. Furthermore, multiple therapeutic cycles of CD/UPRT-armed virus and 5-FC would also increase the synergistic antitumor activity compared with that after a single treatment cycle [31], which was evaluated in this study.

In conclusion, we demonstrated that MDRVV is more highly attenuated in normal cells than the virus harboring the deletion of either gene alone, while retaining its therapeutic replication in PDAC cells that exhibit constitutive ERK1/2 activation in the MAPK pathway. CD/UPRT-armed MDRVV alone efficiently eliminated PDAC, and the antitumor activity was partially enhanced by its combination with 5-FC in vitro and in vivo. Furthermore, the tumor-selective replication of MDRVV was confirmed in PDAC patient-derived tissue cultures. Our findings strongly suggest that regimens comprising the systemic injection of CD/UPRT-armed MDRVV alone and in combination with 5-FC are promising strategies for PDAC treatment.

## Data Availability

The data presented in this study are available in the article and as supplementary material.

## Supplementary Materials

The following are available online at https://www.mdpi.com/article/10.3390/cells10050985/s1, Figure S1: Viral distribution in SCID mice of Figure 2D. Figure S2: Tumor growth and viral distribution in SCID mice bearing peritoneally disseminated BxPC-3 xenografts of Figure 2E. Figure S3: Evaluation of viral LC_50_ with various types of tumor cell lines. Figure S4: In vitro viral oncolytic effect and prodrug combination in other cancer cell lines. Figure S5: Tumor growth before viral treatment in the clinically relevant mouse tumor model of Figure 6. Figure S6: Relationship between in vivo pERK expression and replication of mitogen-activated protein kinase-dependent recombinant vaccinia virus (MDRVV).
