# Supplementary material for: Anti-Tumor Effects of MAPK-Dependent Tumor-Selective Oncolytic Vaccinia Virus Armed with CD/UPRT against Pancreatic Ductal Adenocarcinoma in Mice
Source: Cells. 2021 Apr 23;10(5):985. doi: 10.3390/cells10050985 (PMC8145488; doi:10.3390/cells10050985)
Supplement: Supplementary file 1 [file cells-10-00985-s001.zip › cells-1130052-supplementary.pdf]

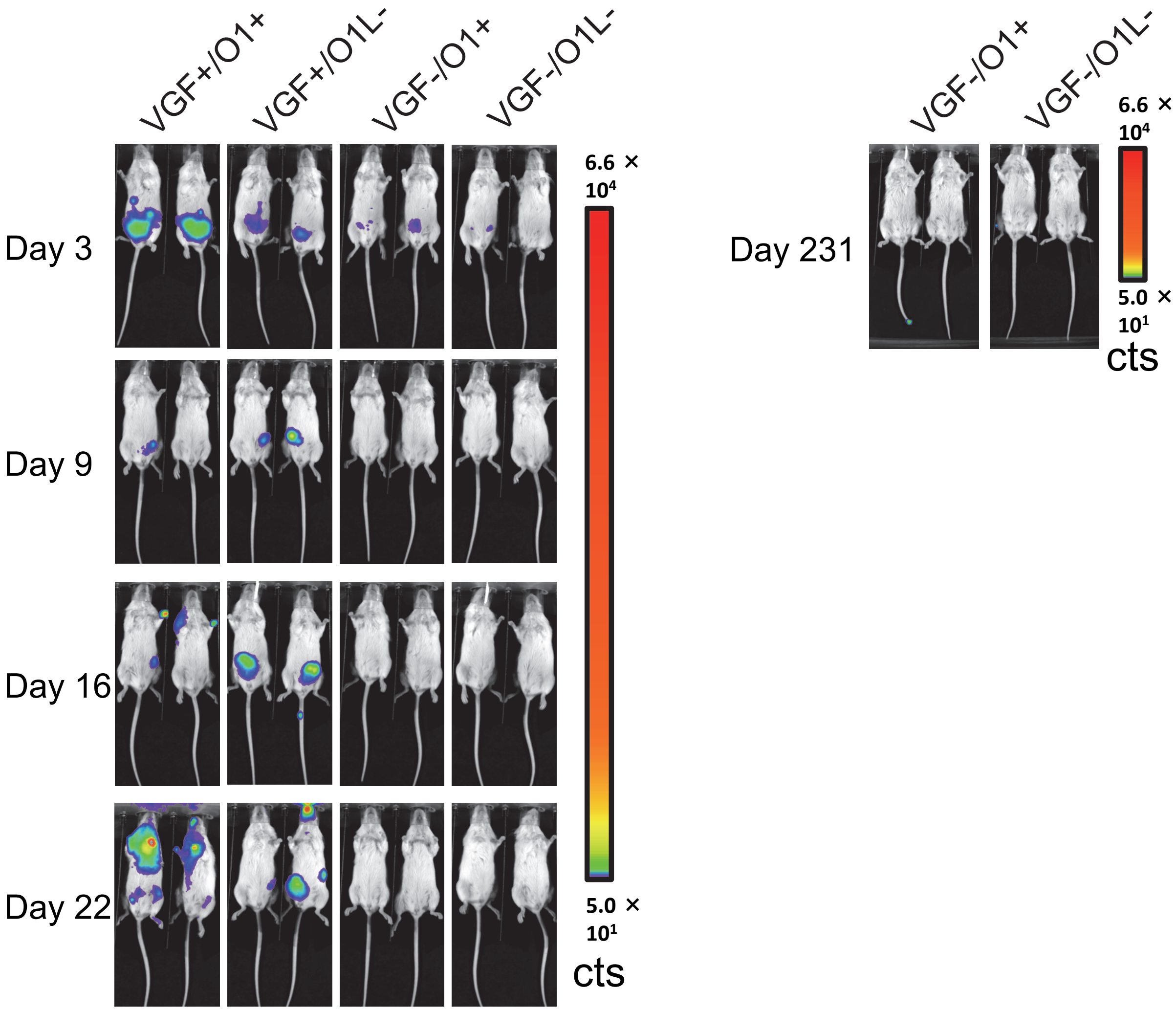

Figure S1. Viral distribution in SCID mice of Figure 2D. SCID mice were injected intraperitoneally with 1 × 10^6 plaque-forming units (PFUs) of each recombinant virus (n=2). Viral replication was non-invasively detected by firefly luciferase (Fluc) luminescence following the I.P. injections of 200 μL VivoGlo Luciferin, In Vivo Grade (3 mg/mouse; Promega, Madison, WI, USA) on days 3, 9, 16, 22, and 231 after viral injection.

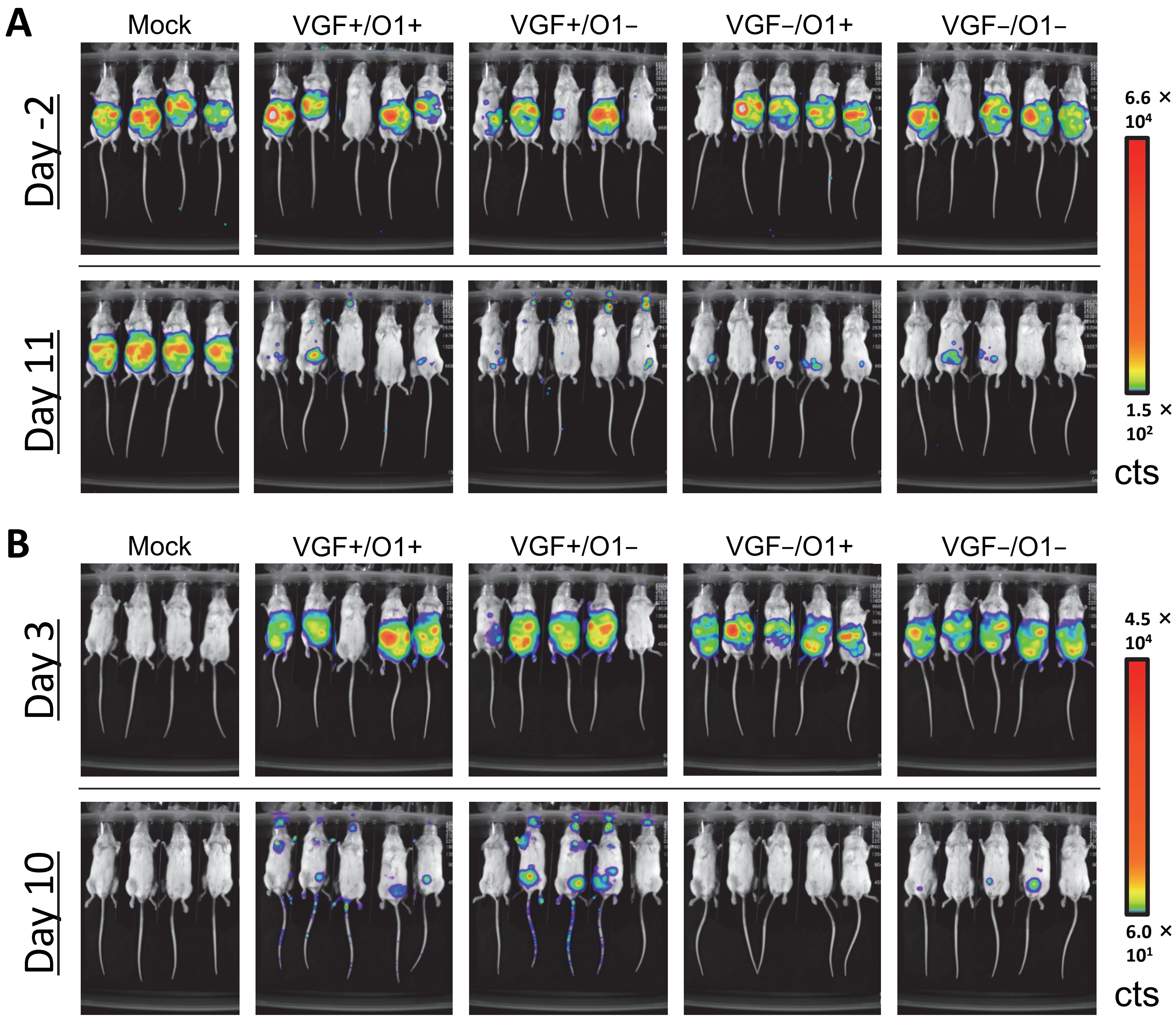

Figure S2. Tumor growth and viral distribution in SCID mice bearing peritoneally disseminated BxPC-3 xenografts of Figure 2E. **(A)** BxPC-3 cells stably expressing Renilla luciferase (5×10^6 cells) were injected intraperitoneally into SCID mice. Eight days after tumor transplantation, the mice received a single intraperitoneal injection of each recombinant virus (1×10^6 PFU). Tumor growth was non-invasively visualized by Renilla luciferase (Rluc) luminescence following the I.P. injections of 150 μL of ViviRen In Vivo Renilla Luciferase Substrate (18.5 μg/mouse; Promega, Madison, WI, USA) 2 day before and 11 days after viral administration. **(B)** Viral replication was non-invasively detected on days 3 and 10 after viral treatment via Fluc luminescence following the I.P. injections of 200 μL VivoGlo Luciferin, In Vivo Grade (3 mg/mouse).

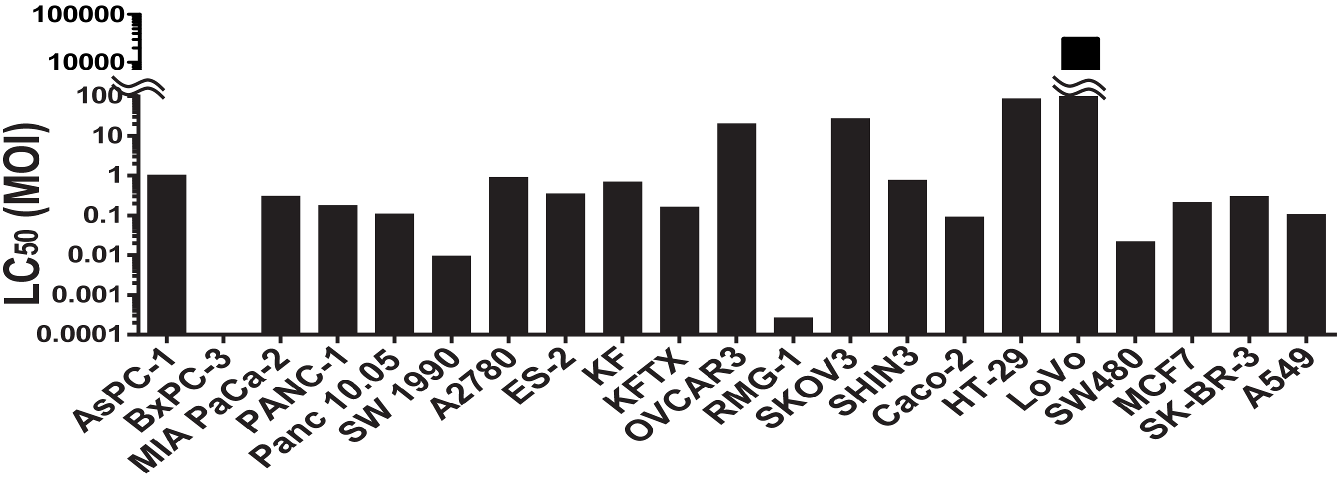

Figure S3. Evaluation of viral LC_{50} with various types of tumor cell lines. Various types of tumor cell lines were infected with mitogen-activated protein kinase (MAPK)-dependent recombinant vaccinia virus (MDRVV; LG-DsRed) at a multiplicity of infection (MOI) of 0, 0.01, 0.1, or 1, and then, 120 h later, cell viability was measured using the CellTiter 96 Aqueous Nonradioactive Cell Proliferation Assay (Promega, Madison, WI, USA). LC_{50} values were calculated from calibration curves of the viability reduction ratio versus virus concentration.

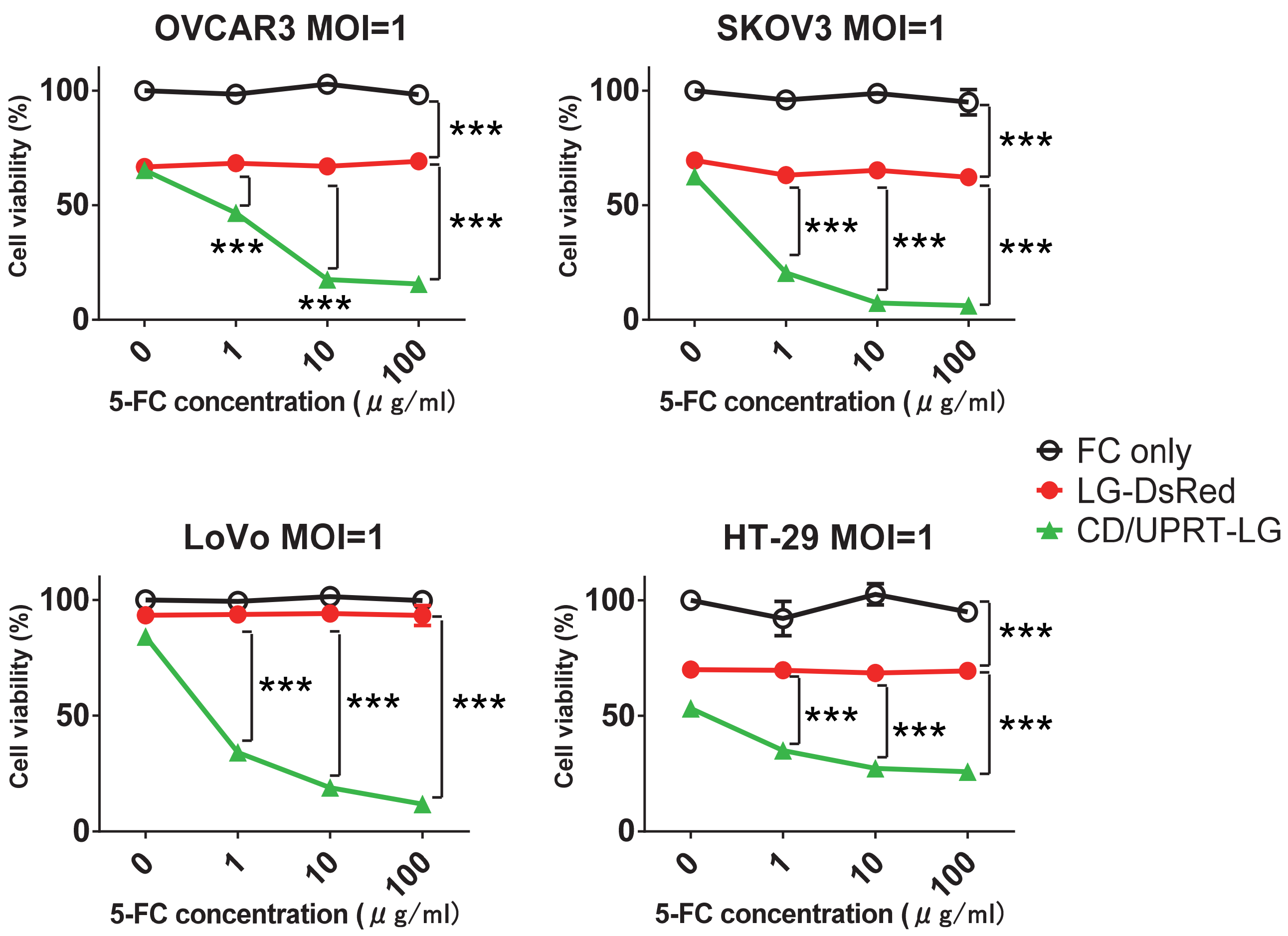

Figure S4. In vitro viral oncolytic effect and prodrug combination in other cancer cell lines. The combined effect of two recombinant viruses (MOI of 1) and 5-FC in other human cancer cell lines (having higher viral LC_{50} values) as described in Figure S3. The data are presented as the mean ± SD (n = 3). ***$P < 0.001$ (two-tailed unpaired t-test).

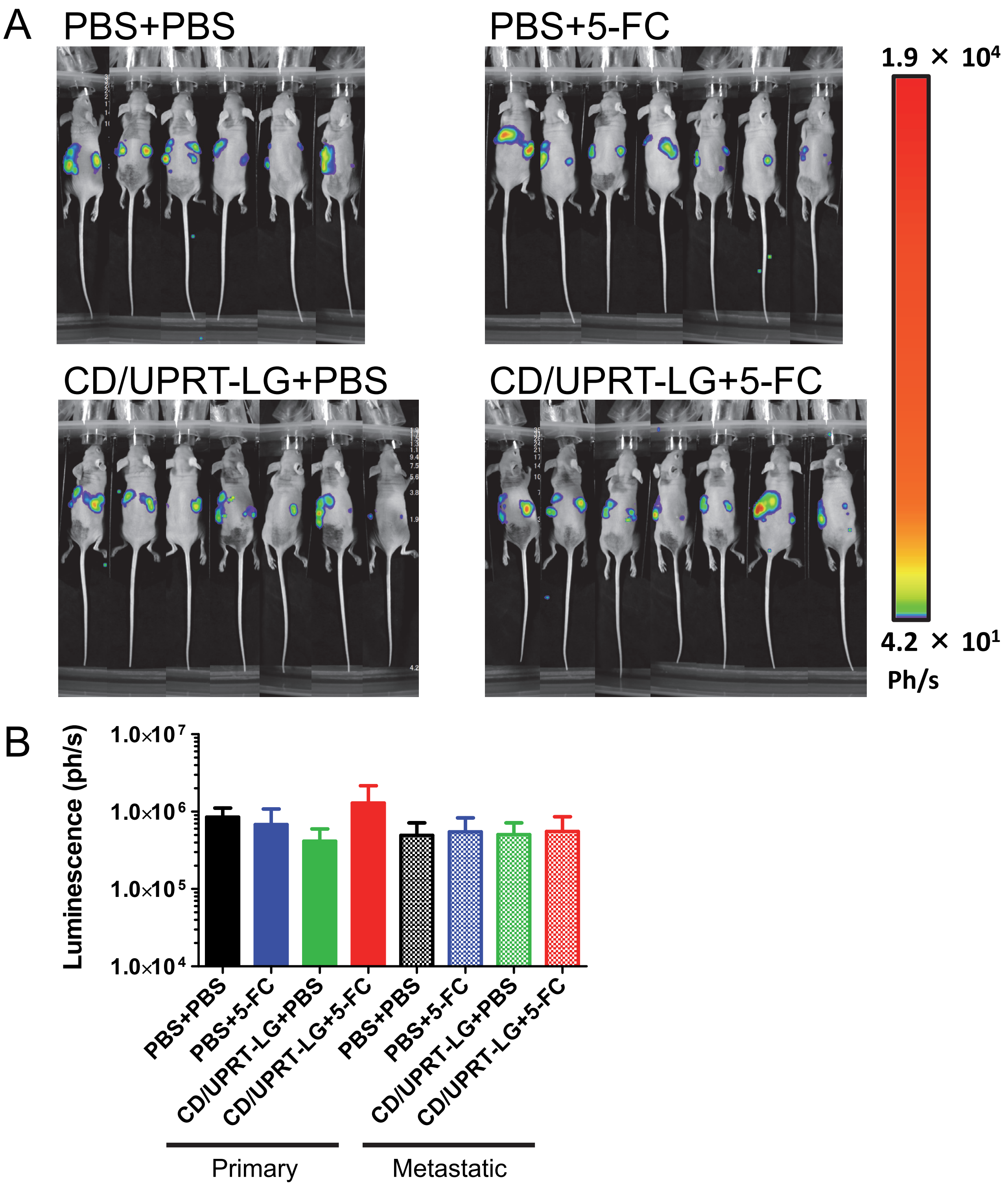

Figure S5. Tumor growth before viral treatment in the clinically relevant mouse tumor model of Figure 6. **(A)** Tumor growth in primary spleens and liver metastasis was non-invasively detected via Rluc luminescence following the I.P. injections of 150 μL of ViviRen In Vivo Renilla Luciferase Substrate (18.5 μg/mouse) on day 1 before viral treatment. **(B)** Quantification of tumor Rluc luminescence was determined from (A). The data are presented as the mean + SD (n = 6–7).

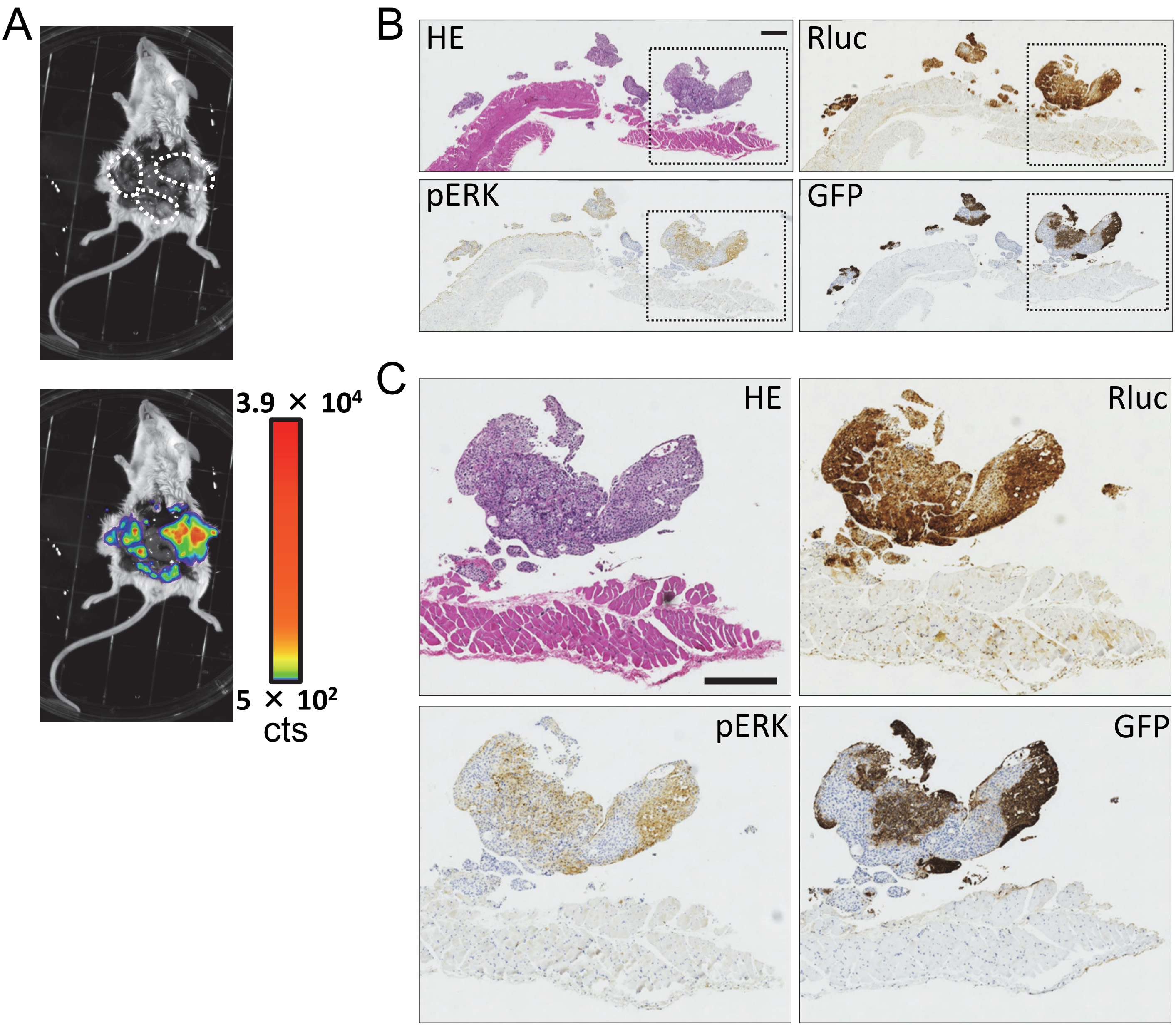

Figure S6. Relationship between in vivo pERK expression and viral replication of MDRVV. **(A)** BxPC-3 cells stably expressing Renilla luciferase (5 × 10^6 cells) were injected intraperitoneally into SCID mice. Thirty-three days after tumor transplantation, the mice received a single intraperitoneal injection of LG-DsRed virus (1 × 10^6 PFU). Three days later, the treated mouse was injected intraperitoneally with 200 μL VivoGlo Luciferin, In Vivo Grade (3 mg/mouse), and was sacrificed 15 min after the injection. Immediately, intraperitoneal bright-field (top) and Fluc luminescence (bottom) images were taken with NightSHADE LB985 (Berthold Technologies, Bad Wildbad, Germany). Dotted circles show the tumor sites. **(B)** Subsequently, the tissues were formalin-fixed and paraffin-embedded, sliced and stained with hematoxylin and eosin, an anti-GFP antibody (CST, Tokyo, Japan), an anti-phosphorylated p44/42 MAPK protein (Erk1/2) antibody (CST, Tokyo, Japan), or anti-Rluc antibody (MBL, Nagoya, Japan). Scale bar, 300 μm. **(C)** Extended images of those marked with squares in (B). Scale bar, 300 μm.
